# Predictors of hyperlactataemia among children presenting with malaria in a low transmission area in The Gambia

**DOI:** 10.1186/1475-2875-12-423

**Published:** 2013-11-15

**Authors:** Krishnan Bhaskaran, Augustine O Ebonyi, Brigitte Walther, Michael Walther

**Affiliations:** 1 Department of Non-communicable Diseases Epidemiology, London School of Hygiene and Tropical Medicine, London, UK; 2 MRC Laboratories (UK) The Gambia Unit, Fajara, The Gambia; 3 Laboratory of Malaria Immunology & Vaccinology, Division of Intramural Research, National Institute of Allergy and Infectious Diseases, National Institutes of Health, Rockville, MD, USA

**Keywords:** Hyperlactataemia, Lactate, Malaria, Children

## Abstract

**Background:**

Hyperlactataemia and metabolic acidosis are important risk factors for malaria death, but measuring lactate at the point of care is not financially viable in many resource-poor settings. This study aimed to identify combinations of routinely available parameters that could identify children at high risk of hyperlactataemia.

**Methods:**

Using data from a study of Gambian children aged six months to 16 years with severe or uncomplicated malaria, logistic regression modelling with a forward stepwise model selection process was used to develop a predictive model for hyperlactataemia from routinely available demographic, clinical and laboratory parameters. Potential predictors of hyperlactataemia considered for the modelling process were patient characteristics (age, sex, prior use of anti-malarials, and weight percentile for age), respiratory symptoms (deep breathing, irregular respiration, use of accessory muscles of respiration, lung crepitations, grunting respiration, cough, and age-specific respiratory rate), other clinical parameters recorded at presentation (duration of symptoms, Blantyre coma score, number of convulsions prior to admission, axillary temperature, dehydration, severe prostration, splenomegaly) and laboratory measures from blood tests (percentage parasitaemia, white cell count, lymphocyte count, neutrophil count, monocyte count, platelet count, haemoglobin level, blood glucose level).

**Results:**

495 children were included, and 68 (14%) had laboratory-confirmed hyperlactataemia (lactate > 7 mmol/L). Four features were independently associated with increased hyperlactataemia risk in a multivariable age- and sex-adjusted model: lower Blantyre score (odds ratio (OR) compared to score 5 = 2.68 (95% CI, 1.03-6.96) for score 3–4 and 6.18 (95% CI, 2.24-17.07) for score 0–2, p = 0.001), higher percentage parasitaemia (OR = 1.07 (1.03-1.11) per 0031% increase, p < 0.001), high respiratory rate for age (OR = 3.09 (1.50-6.38) per unit increase, p = 0.002), and deep breathing (OR = 2.81 (1.20-6.60), p = 0.02). Cross-validated predictions from the final model achieved area under the receiver operating characteristic curve of 0.83.

**Conclusions:**

This study identified predictors of hyperlactataemia requiring only simple bedside clinical examination and blood film examination that can be carried out in resource-limited settings to quickly identify children at risk of dangerously raised lactate. A simple spreadsheet tool implementing the final model is supplied as supplementary material.

## Background

While *Plasmodium falciparum* malaria declines [1], the proportion of cases with severe malaria may increase [2]. Low-cost tools for early identification of those at highest risk of dying would allow tailoring of limited resources to those who need them most. Metabolic acidosis is one of the most frequent presentations defining severe malaria [3], and is associated with a high risk of death in African children with severe malaria, no matter whether it is captured as base excess [4,5], lactataemia [4,6-10], or by clinical signs of respiratory distress, such as deep breathing or lower chest wall in-drawing [4,7,11].

While deep breathing may reliably detect the most severe acidosis [12] respiratory symptoms are not universal among children with high lactate [13], and in one study, identified only 51% of children with high lactate who died [6], suggesting the usefulness of lactate measurement at the point of care. However, the consumables for lactate measurement devices are expensive and generally unavailable in resource-poor settings [6,14]. It would, therefore, be desirable to have more easily observed clinical and laboratory features that could identify children at risk of high lactate during a malaria episode.

There is mixed evidence regarding correlations of non-respiratory factors that are associated with severe respiratory distress in malaria [9,13,15], and fewer data are available relating hyperlactataemia with combinations of respiratory and non-respiratory clinical factors alongside easily available laboratory measures in a multivariable framework.

The aims of this study were therefore to identify predictors of hyperlactataemia among children with malaria, from a range of clinical and laboratory parameters usually available in relatively resource-poor primary health care settings; to develop a multivariable predictive model that could be used to assess risk for hyperlactataemia; and to implement this in an easy-to-use risk prediction tool.

## Methods

### Study population

The dataset used for these analyses arose from a study into the pathogenesis of severe malaria that was carried out from 2005 to 2011 and enrolled uncomplicated and severe malaria cases in Gambian children aged six months to 16 years [16,17]. Uncomplicated malaria was defined as a history of fever within the previous 48 hours, >5,000 asexual *P. falciparum* parasites/μL (or percentage parasitaemia >0.1%), and no other known cause of fever. In addition to the above, severe malaria required the presence of at least one of the following criteria: haemoglobin (Hb) ≤6 g/dL, blood glucose ≤2.2 mmol/L, repeated convulsions (≥three in previous 24 hours), Blantyre coma score of ≤ two in the absence of hypoglycaemia and persisting for at least two hours, lactate concentration >7 mmol/L (hyperlactataemia), difficulty in breathing (abnormalities in the age-specific respiratory rate, lower chest wall in-drawing or deep breathing), and severe prostration (i e, observed inability to drink or suck in children ≤ six months of age or inability to sit unsupported in those > six months). Cases residing within a 40-km radius south of Banjul and presenting to the health facilities at the Medical Research Council (MRC) in Fajara, the Royal Victoria Teaching Hospital (RVTH) in Banjul, the Jammeh Foundation for Peace (JFP) Hospital in Bundung and the health centre in Brikama were enrolled. Appropriate informed consent was obtained in all cases from parents or legal guardians. The study received ethics approval from the London School of Hygiene and Tropical Medicine ethics committee.

### Outcome and potential explanatory variables

Lactate was measured from a finger-prick blood sample at enrolment using Lactate Pro (Arkray). It was decided *a priori* to use a cut-off of 7 mmol/L for hyperlactataemia in preference over the WHO-recommended 5 mmol/L [3], since the odds of death increase sharply above 7 mmol/L in this study area [7], and mortality in the study cohort was predominantly among children with lactate >7 mmol/L [17]. Of note, there was insufficient power to directly investigate mortality itself as an outcome.

Potential predictors of hyperlactataemia considered for the modelling process were patient characteristics (age, sex, prior use of anti-malarials, and weight percentile for age, derived with reference to Centers for Disease Control age- and sex-stratified growth charts [18]), respiratory symptoms (deep breathing, irregular respiration, use of accessory muscles of respiration, lung crepitations, grunting respiration, cough, and age-specific respiratory rate ratio, defined as respiratory rate/age-specific median respiratory rate [19]), other clinical parameters recorded at presentation (duration of symptoms, Blantyre coma score, number of convulsions prior to admission, axillary temperature, dehydration, severe prostration, splenomegaly) and laboratory measures from blood tests (percentage parasitaemia, white cell count, lymphocyte count, neutrophil count, monocyte count, platelet count, Hb level, blood glucose level). A broad list of candidate variables was considered, which could plausibly be associated either directly with lactate levels, or more generally with malaria severity, on the basis that even variables not causally linked directly to plasma lactate could provide associational information helping to predict risk of high lactate.

### Statistical methods

#### Descriptive statistics and preliminary modelling

Descriptive statistics were initially produced for each potential predictor of hyperlactataemia, by hyperlactataemia status and overall. Associations between potential predictors and hyperlactataemia were then investigated through logistic regression modelling. It was decided *a priori* to include age and sex in all models due to the association of these variables with a wide range of health parameters. Initial preliminary modelling was carried out to examine potential non-linear relationships between continuous variables and hyperlactataemia risk, to examine the usefulness of log-transforming blood count variables, and to decide appropriate choice of categories for multi-category variables. The process and the justification of these decisions are described in detail in Additional file 1. In summary, the preliminary modelling led to the inclusion of age as a linear threshold term above age five years, due to a lack of univariate association with hyperlactataemia at younger ages; the inclusion of blood glucose level as a four-category variable (≤2.2, 2.2-4.4, 4.4-8.3, >8.3 mmol/L) due to an apparently non-linear association with hyperlactataemia; and to the use of log-transformed versions of the total white cell, lymphocyte, neutrophil, monocyte, and platelet count variables. The remaining continuous variables (axillary temperature, age-specific respiratory rate ratio, Hb, percentage parasitaemia) showed no evidence against linearity, so were included in the modelling as simple linear terms. Blantyre score was grouped into scores of 0–2, 3–4 and 5 due to low numbers of children with specific individual scores. History of convulsions was included as a simple binary (yes/no) variable, and duration of symptoms was included in three categories (≤one day, two, ≥three days).

#### Final model selection process

After carrying out the above-described preliminary exploratory analyses to decide on the optimal parametrization of potential predictors of hyperlactataemia, a forward stepwise model selection process was carried out to decide on a final model. At each stage of the model building, every variable was added in turn to the existing model, and the variable with the lowest p-value (based on a Wald test for that variable) was entered into the model providing the p-value was <0.05. After the addition of a new variable, any existing variable in the model no longer significantly associated with the outcome (based on a p-for-exit criterion of 0.10) was dropped (but had the opportunity to re-enter the model in subsequent steps). The model building was done in two stages due to missing data in some variables. Initially, a model was developed restricted to variables that were >90% complete in the dataset, in order to avoid the loss of substantial numbers of children from the analysis. The variables omitted from this first stage were white cell, lymphocyte, neutrophil, monocyte, platelet counts, and splenomegaly. However, a second forward stepwise stage of model building was then carried out among children with complete data on all variables, beginning with the model developed in the first stage, and testing each of the omitted variables for inclusion using the same process as described above.

### Model validity

The predictive performance and validity of the final model were examined using leave-one-out cross-validation, a process that avoids testing the predictive performance of the model using the same data that were used to estimate the model, which could lead to over-optimistic results [20]. Having generated a set of predictions a receiver operating characteristic (ROC) curve was constructed, relating the sensitivity and specificity of the model to the choice of probability cut-off used to define a prediction of hyperlactataemia.

### Sensitivity analysis

To examine sensitivity to the choice of model selection strategy, the model selection was repeated using a backward stepwise instead of forward stepwise process. The model selection procedure was also re-run using the WHO cut-off for hyperlactataemia (>5 mmol/L), to understand whether different predictors would apply when using this definition of hyperlactataemia.

## Results

### Description of study population

Out of a total of 495 children, 68 (14%) had hyperlactataemia, defined as lactate >7 mmol/L. A total of 10 children were known to have died, 8 (12%) in the hyperlactataemia group, and 2 (0.5%) in the non-hyperlactataemia group. The distribution of severe manifestations (hyperlactataemia, severe anaemia, cerebral malaria) is shown by Venn diagram in Additional file 1. The median age of children was five years (IQR 3.3-9.0, Table 1). Children with hyperlactataemia had higher rates of prior anti-malarial use, higher prevalence of respiratory symptoms, impaired consciousness (Blantyre score <5), and severe prostration. There were also apparent differences in laboratory parameters between children with and without hyperlactataemia (Table 2): children with hyperlactataemia had higher median percentage parasitaemia (13.9 *vs* 5.0), lower median platelet counts (55.5 *vs* 104.0 × 10^9^/L), and lower median Hb levels (8.7 *vs* 10.8 g/dL).

**Table 1:** Characteristics of study participants, overall and by hyperlactataemia status

**Characteristics of subjects**	**Hyperlactataemia (blood lactate >7 mmol/L)**	**No hyperlactataemia (blood lactate ≤7 mmol/L)**	**Study population**
*Cell contents below are n (%) unless otherwise stated*			
**Total N**	68 (100.0)	427 (100.0)	495 (100.0)
**Patient characteristics**			
Age in years			
<1	0 (0.0)	3 (0.7)	3 (0.6)
1-4	40 (58.8)	170 (39.8)	210 (42.4)
≥ 5	28 (41.2)	254 (59.5)	282 (57.0)
Median (IQR)	4.0 (3.0 to 6.0)	5.0 (3.3 to 9.0)	5.0 (3.0 to 8.0)
Range	1.0 to 15.0	0.7 to 16.0	0.7 to 16.0
Sex			
Male	30 (44.1)	238 (55.7)	268 (54.1)
Female	27 (39.7)	177 (41.5)	204 (41.2)
*Missing*	11 (16.2)	12 (2.8)	23 (4.6)
Weight %ile for age			
<10	40 (58.8)	231 (54.1)	271 (54.7)
10-24	12 (17.6)	72 (16.9)	84 (17.0)
25-50	6 (8.8)	75 (17.6)	81 (16.4)
50-74	6 (8.8)	28 (6.6)	34 (6.9)
75-89	3 (4.4)	13 (3.0)	16 (3.2)
≥90	1 (1.5)	7 (1.6)	8 (1.6)
*Missing*	0 (0.0)	1 (0.2)	1 (0.2)
Prior use of anti- malarial			
Yes	24 (35.3)	91 (21.3)	115 (23.2)
No	39 (57.4)	315 (73.8)	354 (71.5)
*Missing*	5 (7.4)	21 (4.9)	26 (5.3)
Respiratory symptoms*			
Deep breathing	37 (54.4)	44 (10.3)	81 (16.4)
Irregular respiration	1 (1.5)	1 (0.2)	2 (0.4)
Use of accessory muscles	10 (14.7)	4 (0.9)	14 (2.8)
Lung crepitations	3 (4.4)	9 (2.1)	12 (2.4)
Grunting respiration	7 (10.3)	11 (2.6)	18 (3.6)
Cough	26 (38.2)	158 (37.0)	184 (37.2)
Age specific respiratory rate ratio (median, IQR)	2.3 (1.8 to 2.6)	1.7 (1.5 to 2.1)	1.8 (1.5 to 2.1)
**Other clinical parameters**			
Duration of symptoms in days			
≤ 1	10 (14.7)	34 (8.0)	44 (8.9)
2	35 (51.5)	139 (32.6)	174 (35.2)
3	14 (20.6)	156 (36.5)	170 (34.3)
≥ 4	9 (13.2)	98 (23.0)	107 (21.6)
Median (IQR)	1.0 (1.0 to 2.0)	2.0 (1.0 to 2.0)	2.0 (1.0 to 2.0)
Blantyre coma score			
0-2	27 (39.7)	24 (5.6)	51 (10.3)
3-4	16 (23.5)	46 (10.8)	62 (12.5)
5	24 (35.3)	357 (83.6)	381 (77.0)
*Missing*	1 (1.5)	0 (0.0)	1 (0.2)
Reduced reaction to pain**			
Yes	22 (32.4)	18 (4.2)	40 (8.1)
No	46 (67.6)	409 (95.8)	455 (91.9)
History of Convulsions prior to admission			
0	26 (38.2)	329 (77.0)	355 (71.7)
1	8 (11.8)	42 (9.8)	50 (10.1)
2	9 (13.2)	20 (4.7)	29 (5.9)
≥3	24 (35.3)	32 (7.5)	56 (11.3)
*Missing*	1 (1.5)	4 (0.9)	5 (1.0)
Temperature in °C			
≤37	2 (2.9)	70 (16.4)	72 (14.5)
>37, ≤39	38 (55.9)	235 (55.0)	273 (55.2)
>39	28 (41.2)	122 (28.6)	150 (30.3)
Median (IQR)	38.8 (38.2 to 39.5)	38.4 (37.5 to 39.2)	38.5 (37.6 to 39.2)
Dehydration			
Mild or Moderate	10 (14.7)	43 (10.1)	53 (10.7)
None	58 (85.3)	384 (89.9)	442 (89.3)
Severe prostration			
Yes	54 (79.4)	146 (34.2)	200 (40.4)
No	14 (20.6)	279 (65.3)	293 (59.2)
*Missing*	0 (0.0)	2 (0.5)	2 (0.4)
Splenomegaly			
Yes	11 (16.2)	74 (17.3)	85 (17.2)
No	51 (75.0)	302 (70.7)	353 (71.3)
*Missing*	6 (8.8)	51 (11.9)	57 (11.5)

*All listed respiratory symptoms data were complete, except for one child with missing cough status, and three children with missing respiratory rate (all in the no hyperlactataemia group); age-specific respiratory rate ratio defined as (respiratory rate/median for age).

**Reduced reaction to pain defined as failure to localise pain (i e, flexes to pain or no response to pain).

**Table 2:** Laboratory parameters for study participants, overall and by hyperlactataemia status

**Characteristics of subjects**	**Hyperlactataemia (blood lactate >7 mmol/L)**	**No hyperlactataemia (blood lactate ≤7 mmol/L)**	**Study population**
*Cell contents below are n (%) unless otherwise stated*			
**Total N**	68 (100.0)	427 (100.0)	495 (100.0)
% parasitaemia			
0-9	21 (30.9)	303 (71.0)	324 (65.5)
10-19	26 (38.2)	86 (20.1)	112 (22.6)
20-29	10 (14.7)	20 (4.7)	30 (6.1)
≥ 30	10 (14.7)	6 (1.4)	16 (3.2)
*Missing*	1 (1.5)	12 (2.8)	13 (2.6)
Median (IQR)	13.9 (7.1 to 25.0)	5.0 (2.0 to 10.0)	6.0 (2.0 to 12.4)
Total white blood cells (x10^9^/L)			
Median (IQR)	9.6 (7.0 to 13.5)	8.0 (6.1 to 10.8)	8.2 (6.2 to 11.3)
*Missing [N(%)]*	17 (25.0)	50 (11.7)	67 (13.5)
Total lymphocytes (x10^9^/L)			
Median (IQR)	2.7 (2.1 to 4.4)	2.0 (1.3 to 3.0)	2.1 (1.4 to 3.1)
*Missing [N(%)]*	17 (25.0)	62 (14.5)	79 (16.0)
Total neutrophils (x10^9^/L)			
Median (IQR)	5.5 (4.0 to 8.2)	5.1 (3.6 to 7.4)	5.2 (3.6 to 7.5)
*Missing [N(%)]*	17 (25.0)	116 (27.2)	133 (26.9)
Total monocytes (x10^6^/L)			
Median (IQR)	649.8 (466.1 to 1011.2)	525.3 (366.1 to 798.1)	537.4 (377.6 to 813.2)
*Missing [N(%)]*	18 (26.5)	115 (26.9)	133 (26.9)
Total platelets (x10^9^/L)			
Median (IQR)	55.5 (28.0 to 86.0)	104.0 (56.0 to 161.0)	95.0 (51.0 to 158.0)
*Missing [N(%)]*	18 (26.5)	40 (9.4)	58 (11.7)
Hb (g/dL)			
Median (IQR)	8.7 (6.3 to 10.5)	10.8 (8.9 to 12.2)	10.5 (8.7 to 12.1)
*Missing [N(%)]*	3 (4.4)	14 (3.3)	17 (3.4)
Blood glucose (mmol/L)			
≤2.2	7 (10.3)	7 (1.6)	14 (2.8)
>2.2, ≤4.4	13 (19.1)	39 (9.1)	52 (10.5)
>4.4, ≤8.3	29 (42.6)	298 (69.8)	327 (66.1)
>8.3	18 (26.5)	80 (18.7)	98 (19.8)
*Missing [N(%)]*	1 (1.5)	3 (0.7)	4 (0.8)
Median (IQR)	6.8 (4.2 to 8.6)	6.5 (5.4 to 7.8)	6.5 (5.3 to 7.9)

Note: Missing values for full blood count parameters are in part explained by the fact that there was no facility to perform full blood counts for children enrolled in the first year of the study (n = 31).

### Age/sex-adjusted associations of individual variables with hyperlactataemia

The analysis was initially restricted to variables that were >90% complete in the dataset, resulting in 407 children being included in the first phase of modelling. In logistic models for hyperlactataemia, adjusted only for age and sex, deep breathing, use of accessory muscles, grunting respiration, higher age-specific respiratory rate ratio, shorter duration of symptoms, lower Blantyre coma score, history of convulsions, severe prostration, higher parasitaemia and lower Hb were all strongly associated with higher risk of hyperlactataemia (Table 3). Blood glucose was also strongly associated with hyperlactataemia risk, with the lowest risk among those with blood glucose between 4.4 and 8.3 mmol/L, and higher risk at lower and higher values.

**Table 3:** Age/sex-adjusted associations of each variable with hyperlactataemia

**Variable**	**Odds ratio and 95% CI**	p-value*
Weight %ile for age		0.83
<10	1.00 (REF)	
10-24	0.95 (0.42 to 2.14)	
25-50	0.50 (0.19 to 1.37)	
50-74	1.02 (0.32 to 3.22)	
75-89	1.31 (0.26 to 6.44)	
≥90	0.75 (0.09 to 6.33)	
Prior use of anti-malarial	1.71 (0.88 to 3.30)	0.11
Deep breathing	8.46 (4.22 to 16.96)	<0.001
Irregular breathing	5.94 (0.36 to 98.49)	0.21
Use of accessory muscles	11.51 (2.58 to 51.26)	0.001
Lung crepitations	2.48 (0.64 to 9.70)	0.19
Grunting respiration	4.78 (1.56 to 14.60)	0.006
Cough	1.02 (0.54 to 1.90)	0.96
Age-specific respiratory rate ratio** (per unit increase)	5.24 (2.75 to 9.98)	<0.001
Duration of symptoms (days)		0.003
≤1	1.00 (REF)	
2	0.67 (0.28 to 1.62)	
≥3	0.24 (0.09 to 0.63)	
Blantyre coma score		<0.001
5	1.00 (REF)	
3-4	4.35 (1.83 to 10.35)	
0-2	15.12 (6.42 to 35.62)	
Any convulsions	5.36 (2.74 to 10.49)	<0.001
Axillary temperature (per °C increase)	1.40 (1.03 to 1.92)	0.03
Dehydration	1.56 (0.67 to 3.62)	0.3
Severe prostration	5.67 (2.72 to 11.81)	<0.001
Splenomegaly	0.86 (0.38 to 1.97)	0.72
% parasitaemia (per 1% increase)	1.09 (1.05 to 1.12)	<0.001
Hb (per 1 g/dL increase)	0.78 (0.69 to 0.89)	<0.001
Blood glucose (mmol/L)		0.003
≤2.2	1.00 (REF)	
>2.2, ≤4.4	0.22 (0.05 to 1.04)	
>4.4, ≤8.3	0.09 (0.02 to 0.37)	
>8.3	0.17 (0.04 to 0.76)	

*p-values are from Wald tests for each parameter in models adjusted only for age (linear term for number of years since age five years) and sex.

**Age-specific respiratory rate ratio defined as (respiratory rate/median for age).

Note: models were fitted among the 407 children with non-missing data for all the variables in the Table.

### Development of multivariate predictive model for hyperlactataemia

The forward stepwise selection process, with age and sex included as *a priori* (“forced”) variables, generated a final model containing Blantyre score, percentage parasitaemia, age-specific respiratory rate ratio, and presence of deep breathing (Table 4). None of the variables omitted from the first phase of modelling due to missing data were added to the model in the second phase of modelling. The area under the ROC curve for the final model was 0.85, using the full dataset (see below for cross-validated model performance statistics). In the final model, lower Blantyre score, higher parasitaemia, higher age-specific respiratory rate ratio, and deep breathing were all strongly associated with increased probability of hyperlactataemia (Table 5). A Microsoft Excel spreadsheet implementing the final model to predict the probability of hyperlactataemia based on these factors was produced (Additional file 2 - available as web-only supplementary material - note that this spreadsheet tool can also be opened in the open source spreadsheet application OpenOffice Calc).

**Table 4:** Forward stepwise selection of predictive model for hyperlactataemia

**Model**	**p-value for additional variable**	**Akaike information criterion**	**Area under the receiver operating characteristic (ROC) curve**
Age + sex		291.6	0.6
Age + sex + Blantyre score	1.7 × 10-09	254	0.76
Age + sex + Blantyre score +% parasitaemia	8.2 × 10-06	236.1	0.83
Age + sex + Blantyre score +% parasitaemia + age-specific respiratory rate ratio	5.0 × 10-04	225.6	0.84
Age + sex + Blantyre score +% parasitaemia + age-specific respiratory rate ratio + deep breathing	0.018	222.2	0.85

Note: models were fitted among the 407 children with non-missing data for all the variables in the initial model selection process (see Methods).

**Table 5:** Final model for hyperlactataemia

	**Estimated OR and 95% CI**		p-value*
Age (per year above 5)	0.98	(0.83 to 1.15)	0.81
Male sex	1.43	(0.67 to 3.04)	0.35
Blantyre score			0.001
5	1.00 (REF)		
3-4	2.68	(1.03 to 6.96)	
0-2	6.18	(2.24 to 17.07)	
% parasitaemia (per 1% increase)	1.07	(1.03 to 1.11)	<0.001
Age-specific respiratory rate ratio (per unit increase)	3.09	(1.50 to 6.38)	0.002
Deep breathing	2.81	(1.20 to 6.60)	0.018

Note: the predicted odds of hyperlactataemia for an individual child based on the final model are as follows:

log odds of HL = −5.852724 - 0.0198901*(years since fifth birthday if aged < five years, 0 otherwise) + 0.3589414 (if male sex) + 0.9853369 (if Blantyre score is 3 or 4) + 1.821678 (if Blantyre score is 0, 1, or 2) + 0.0664418*(% parasitaemia) + 1.129665*(age-specific respiratory rate ratio) +1.033156 (if deep breathing is present).

The predicted probability of HL is calculated as Pr(HL) = e^(predicted log odds)^/(1 + e^(predicted log odds)^).

### Model validation

**Figure 1:**
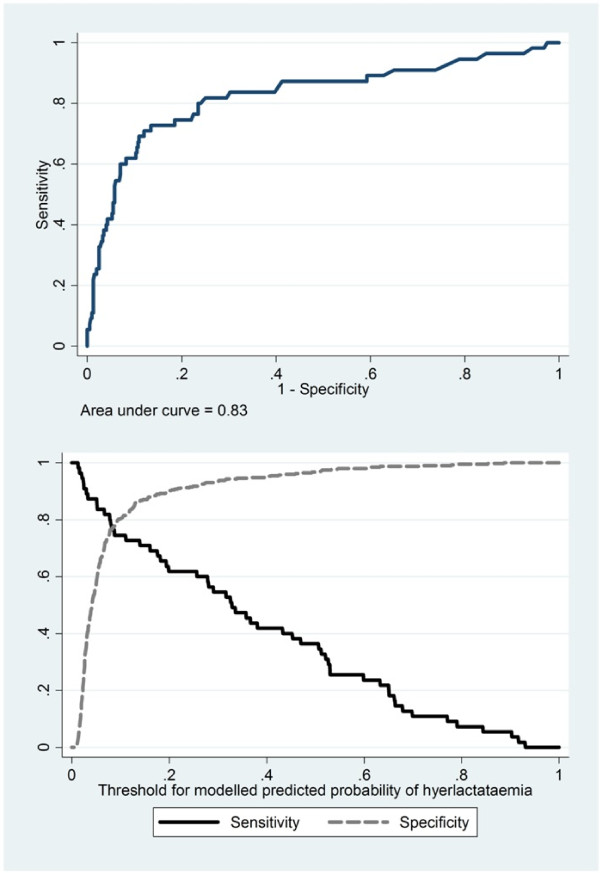
Cross-validated receiver operating characteristic (ROC) curve for the final model, and sensitivity and specificity by predicted probability cut-off.

Figure 1 shows the ROC curve and sensitivity/specificity of the final predictive model. These curves were estimated using leave-one-out cross validation to avoid assessing model performance on the same data that were used to generate the model. The area under the ROC curve was 0.83. Sensitivity and specificity naturally were dependent on choice of the threshold value chosen. At the crossover point (equal sensitivity and specificity), the sensitivity and specificity met at a value of 76%.

### Sensitivity analysis

Using a backward rather than forward stepwise model selection strategy produced a similar model but included two extra terms, namely glucose category and Hb level. As in the main analysis, the model also included Blantyre score, percentage parasitaemia, age-specific respiratory rate ratio, and deep breathing. The model appeared to perform similarly (cross-validated area under ROC curve = 0.82, compared with 0.83 for the model from the main analysis). When the model selection procedure was re-run using the WHO cut-off for hyperlactataemia (>5 mmol/L), a slightly different set of parameters were selected (prostration, percentage parasitaemia, Blantyre score, grunting, and age-specific respiratory ratio) but a similar level of predictive performance was achievable (cross-validated area under ROC curve = 0.82).

## Discussion

In a study of 495 Gambian children with uncomplicated or severe malaria, four features were identified that were independently associated with an increased risk of hyperlactataemia in a multivariable age- and sex-adjusted model: lower Blantyre score, higher percentage parasitaemia, high respiratory rate for age, and presence of deep breathing. Cross-validated predictions from the final model achieved area under the receiver operating characteristic curve of 0.83, while the sensitivity and specificity curves met at a value of 76%, though in practice, clinicians might prioritize higher sensitivity at the expense of lower specificity, given the potential implications of missing a diagnosis of hyperlactataemia. The final model was implemented in a simple spreadsheet that predicts risk of hyperlactataemia given the above factors (available as supplementary online material).

Each of the variables included in the final model are plausible in the context of the pathophysiology and epidemiology of severe malaria. A higher respiratory rate and deep breathing are both compensatory pathophysiologic mechanisms for metabolic acidosis of which hyperlactataemia is a major contributor, especially in children [21,22]. The association of lower Blanytre score (impaired consciousness) with hyperlactataemia is consistent with the observation that cerebral malaria and respiratory distress often occur together [5,11]. This results in markedly increased mortality [5,11], and is associated with particularly high lactate levels [17], essentially indicating a major organ dysfunction [22]. At high parasite burden, parasite glycolysis contributes to hyperlactataemia and hypoglycaemia, in addition to the major contribution from increased glucose consumption, plus reduced gluconeogenesis in the human host [21]. More broadly, the predictors identified all suggest a more severe presentation of disease, so it is expected that these would correlate with hyperlactataemia, which also tends to feature in more severe cases [10,23].

There has been little work done to date on identifying predictors of hyperlactataemia among children with malaria. In line with the results of the present study, Mtove *et al*. [6] found high parasitaemia, deep breathing (also included in the main model from the present analysis), hypoglycaemia and low Hb (terms that were included in the model described in this paper when a backward stepwise approach was performed) to be strongly associated with lactate levels >5 mmol/L. Newton *et al*. investigated whether a range of clinical and laboratory parameters predicted lactate levels (as a continuous measure) among children in three hospitals in Malawi, Kenya and Ghana [13]. Although the authors found that deep and/or irregular breathing, coma and hypoglycaemia were associated with hyperlactataemia across all sites, the adjusted-R^2^ values from multivariable models, including all candidate predictors, did not exceed 0.42, and the authors commented that the predictors they had identified were not able to reliably identify children with hyperlactataemia or acidosis. In contrast the final model from the present study appears from cross-validation to have comparatively strong predictive performance. This may be explained by the different parametrizations of variables– for example, parasitaemia and respiratory rate for age were used as continuous variables here, in contrast with the binary hyperparasitaemia and irregular breathing variables used by Newton *et al*.; alternatively, it may be that in the different setting of that study, and given the different characteristics of patients and health care facilities, lactate was simply less strongly associated with other observed parameters than in the present study, or that by Mtove *et al*. [6]. The inclusion of percentage parasitaemia in the final model presented here contrasts with a study in Uganda, in which prevalence of respiratory distress was not found to differ across regions with different average parasite loads [15]. However, this ecological comparison may have missed individual-level associations; the authors also commented that there may have been some misclassification between malaria with respiratory distress and pneumonia. In the present study, a relatively high cut-off of peripheral parasitaemia was used for inclusion (which improves the specificity of diagnosis of malaria) [24], and no evidence was found of bacterial co-infection using specific PCR for the two most common bacterial pathogens, *Streptococcus pneumoniae* and non-typhoid *Salmonella*[25], therefore such misclassification is unlikely to have been an important issue.

Historically, individual symptoms such as deep breathing and other respiratory observations have been used as surrogates or warning signals for hyperlactataemia and acidosis, though a study by Crawley *et al.* suggesting four distinct respiratory patterns in cerebral malaria suggested a need for more training to identify telltale combinations of respiratory signs [26]. In keeping with an earlier study [13], a large proportion of children with measurable hyperlactaemia in the present study did not have deep breathing recorded (31/68, 46%), and only a single child had irregular breathing. Other single-dimension predictors were equally unhelpful – only 27/68 children with hyperlactataemia (40%) had diagnosed cerebral malaria. The model makes better use of all the available information on a particular child by combining information from a number of predictors, both respiratory and non-respiratory, and so, for example, could flag up an increased risk of hyperlactaemia in a child with high parasitaemia and/or a reduced Blantyre score, but no respiratory symptoms, where this risk might otherwise have been missed. The spreadsheet tool provided alongside this paper demonstrates that the model can be implemented easily in a clinical situation with only basic computing facilities available, though further validation work is recommended before the model is put to broad use. Ultimately, it is hoped that this model would allow the quick and easy identification of children at risk of hyperlactataemia, enabling early prioritization and treatment of such children and a consequent reduction in the risk of severe complications and death.

This study has a number of limitations. The sample size was relatively small, so there may have been insufficient power to detect relatively weak associations between candidate predictors and hyperlactataemia, and some variables may have been omitted that could improve predictive performance. That said, this apparent drawback may have led to improved model parsimony since any variables omitted for this reason are likely to have only weakly contributed to predicting hyperlactataemia risk, and from a practical perspective there is a premium on parsimony since a predictive tool requiring a large number of inputs would be difficult to use. The data came from a single West African country where malaria transmission is comparatively low [27]. Considering that the predominant clinical presentation of severe malaria differs according to endemicity, other clinical parameters maybe more suited to predict lactate in high transmission areas. Due to the relatively small sample size, there was insufficient power to directly model death as an outcome; a worthwhile area of future research using larger datasets would be to develop models relating clinical and laboratory parameters similar to those included in the present study to mortality risk.

How pronounced a symptom is at presentation is in part determined by factors such as ease of access to medical facilities. The model may thus not perform as well in other settings. A further issue is that validation of the predictive model was carried out within the same Gambian setting used to develop it. To reduce the problems associated with this, leave-one-out cross validation was used so that at no point was the model’s performance tested on a data point used in the model fitting, which could have led to over-optimistic results [20]. Nevertheless, until the model is tested in a broader range of settings, which is to be encouraged, there can only be limited confidence about its generalizability.

Despite these limitations, the detailed data recorded for the original study upon which this analysis was based enabled associations between a broad range of clinical and laboratory characteristics and measured lactate to be assessed, with all variables measured objectively by trained health care workers. Extensive preliminary modelling was carried out to characterize associations between candidate predictors and hyperlactataemia, including allowance for non-linearity; a systematic analytic strategy was then employed, and the sensitivity of results to the choice of model selection process was checked.

## Conclusions

Hyperlactataemia is among the most important risk factors for malaria death, but measuring lactate or metabolic acidosis requires the use of blood lactate machines or blood gas analysers which are expensive and often beyond the financial reach of health facilities in resource-poor settings that care for children with potentially life-threatening malaria. The predictors of hyperlactataemia identified in this study require only simple bedside clinical examination (Blantyre coma score, respiratory rate and deep breathing) and blood film examination for malaria parasites, both of which can be easily carried out in a wide range of settings, and not only by doctors but also community health workers. The final predictive model was implemented in a spreadsheet to produce a simple user-friendly risk prediction tool, which could be used in clinical practice to quickly identify children at risk of dangerously raised lactate and in need of further investigations and treatment. It is recommended that the model be tested and validated in a variety of settings to confirm its utility.

## Competing interests

The authors declare that they have no competing interests.

## Authors’ contributions

KB, AE and MW designed the study. BW contributed to the data collection, processing and cleaning. KB did the statistical analysis. KB and AE wrote the first draft. All authors commented on subsequent drafts and approved the final manuscript.

## Supplementary Material

Additional file 1 **Web Appendix v6.1.pdf.** Web appendix containing more detailed description of the preliminary modelling process, and an extra descriptive figure. Click here for file

Additional file 2 **HL Model Based Risk Prediction Tool v5.1.xlsx.** Spreadsheet implementation of final predictive model. Click here for file
