# Supplementary material for: Predictors of hyperlactataemia among children presenting with malaria in a low transmission area in The Gambia
Source: Malar J. 2013 Nov 15;12:423. doi: 10.1186/1475-2875-12-423 (PMC3842629; doi:10.1186/1475-2875-12-423)
Supplement: Additional file 1 — Web Appendix v6.1.pdf. Web appendix containing more detailed description of the preliminary modelling process, and an extra descriptive figure. [file 1475-2875-12-423-S1.docx]

**Web Appendix**

*Detailed description of preliminary modelling process*

To allow for a possible non-linear association between age and hyperlactataemia risk, we initially examined models for age using first a 3-knot natural cubic spline, and then age categories. This preliminary modelling suggested little association between age and hyperlactataemia up to age 5 years, and then an inverse linear relationship; for all subsequent models, age was therefore included as a linear threshold term from age 5, taking the value 0 for children aged up to age 5 years, and (age-5) for children aged ≥5 years; this parametrisation effectively omits age as a predictor for those aged under 5 as suggested by the preliminary modelling. Other choices of age threshold were considered but the choice of 5 years was the best fit (based on minimising the Akaike Information Criterion (AIC) of the model). Before commencing the main model selection process, we similarly examined potential non-linearity for all other continuous variables (adjusting for age and sex), and we also examined log transformations for blood count variables; our decisions on how to include variables in the final model selection process were based on Wald tests for non-linearity, and minimisation of the AIC in candidate models. From this process we decided to include blood glucose level in the modelling as a 4-category variable (≤2.2, 2.2-4.4, 4.4-8.3, >8.3 mmol/L) due to an apparently non-linear association with hyperlactataemia (the use of these categories resulted in lower AIC spline-based or quadratic paramaterization: AIC=325.7 for categories, 329.4 for a spline model, 334.1 for a quadratic model), and to use log-transformed versions of the total white cell, lymphocyte, neutrophil, monocyte, and platelet count variables. For the remaining continuous variables (axillary temperature, age-specific respiratory rate ratio, Hb, % parasitaemia) there was no evidence against linearity, so we included these variables in the modelling as simple linear terms.

We then considered the optimal choice of categories for multi-category variables. Blantyre score was initially grouped into scores of 0-2, 3-4 and 5 due to low numbers of children with specific individual scores. In an age/sex-adjusted model, there was evidence that the odds of hyperlactataemia differed between the 0-2 and 3-4 categories, therefore all 3 categories were retained for the final model selection.

Preliminary modelling also suggested that number of convulsions prior to admission was no better a predictor of hyperlactataemia than a binary variable denoting presence of any convulsions, therefore, history of convulsions (yes/no) was taken forward for the final model selection. Finally, four duration of symptoms categories (≤1 day, 2, 3, ≥4 days) were examined and reduced to three categories for subsequent modelling (≤1 day, 2, ≥3 days) since a Wald test suggested no evidence of a difference in hyperlactataemia risk in the 3 and ≥4 days categories.

*Appendix Figure A1: Venn diagram showing the distribution of severe malaria syndromes among the 495 children included*
